# Silencing HOXD10 by promoter region hypermethylation activates ERK signaling in hepatocellular carcinoma

**DOI:** 10.1186/s13148-017-0412-9

**Published:** 2017-10-23

**Authors:** Yulin Guo, Yaojun Peng, Dan Gao, Meiying Zhang, Weili Yang, Enqiang Linghu, James G. Herman, François Fuks, Guanglong Dong, Mingzhou Guo

**Affiliations:** 1 Department of Gastroenterology and Hepatology, Chinese PLA General Hospital, #28 Fuxing Road, Beijing, 100853 China; 2 Department of General Surgery, Chinese PLA General Hospital, #28 Fuxing Road, Beijing, 100853 China; 3 Medical College of NanKai University, #94 Weijin Road, Tianjin, 300071 China; 4 The Hillman Cancer Center, University of Pittsburgh Cancer Institute, 5117 Centre Avenue, Suite 2.18/Research, Pittsburgh, PA 15213 USA; 5 Laboratory of Cancer Epigenetics, Free University of Brussels (U.L.B.), 808 Route de Lennik, 1070 Brussels, Belgium

**Keywords:** HOXD10, DNA methylation, Hepatocellular carcinoma, IGFBP3, ERK1/2

## Abstract

**Background:**

Hepatocellular carcinoma is the fifth most common malignancy and the third leading cause of cancer-related death worldwide. Dysregulation of HomeoboxD10 (HOXD10) was found to suppress or promote cancer progression in different cancer types. The function and regulation of HOXD10 remain unclear in human hepatocellular carcinoma (HCC).

**Methods:**

Primary HCC samples (117), normal liver tissue samples (15), and 13 HCC cell lines (SNU182, SNU449, HBXF344, SMMC7721, Huh7, HepG2, LM3, PLC/PRF/5, BEL7402, SNU387, SNU475, QGY7703, and Huh1) were included in this study. Methylation-specific PCR, flow cytometry, western blot, transwell, siRNA, and chromatin immunoprecipitation assays were employed.

**Results:**

HOXD10 was methylated in 76.9% (90/117) of human primary HCC samples. HOXD10 methylation was significantly associated with vessel cancerous embolus, tumor cell differentiation, and the 3-year overall survival rate (all *P* < 0.05). The expression of HOXD10 was regulated by promoter region methylation. HOXD10 suppressed colony formation, cell proliferation, cell invasion and migration, and induced G2/M phase arrest and apoptosis in HCC cells. HOXD10 suppressed HCC cell xenograft growth in mice. HOXD10 suppresses HCC growth by inhibiting ERK signaling.

**Conclusion:**

HOXD10 is frequently methylated in human HCC, and the expression of HOXD10 is regulated by promoter region methylation. HOXD10 suppresses HCC cell growth both in vitro and in vivo. HOXD10 suppresses human HCC by inhibiting ERK signaling.

**Electronic supplementary material:**

The online version of this article (10.1186/s13148-017-0412-9) contains supplementary material, which is available to authorized users.

## Background

Hepatocellular carcinoma (HCC) is the fifth most common malignancy and the third leading cause of cancer-related death worldwide [1]. In China, HCC is the fourth most commonly diagnosed cancer in men, and it is the third leading cause of cancer death for both men and women [2]. The 5-year survival rate remains below 12% [3]. The mechanisms underlying the development and progression of HCC remain unclear. Chromosomal amplifications (1*q*, 6*p*, 8*q*, 17*q*, and 20*q*) and deletions (4*q*, 8*p*, 11*q*, 13*q*, 16*q*, and 17*p*) are frequent events in HCC [4]. In 5–10% of HCC patients, high-level amplifications have been described in 6*p*21 (vascular endothelial growth factor A, *VEGFA*) and 11*q*13 (cyclin D1, *CCND1*) [5]. Accumulating evidence has shown that epigenetic as well as genetic alterations play important roles in the development of many cancers [6–8]. Epigenetic inactivation of tumor suppressor genes has been frequently found in HCC [9]. Homeobox (*HOX*) genes encode homeoproteins, which share a common homeodomain and serve as important transcription factors targeting downstream proteins [10]. Homeoproteins play an important role in development and carcinogenesis by modulating cell growth, migration, cell cycle, and apoptosis [11–14]. Homeobox D10 (HOXD10) is a member of the homeobox gene family. HOXD10 expression levels and functions vary by cancer type [14–17]. In this study, we analyzed the regulation and the function of HOXD10 in human HCC.

## Methods

### Human tissue samples and cell lines

Primary human hepatocellular carcinoma samples (117 cases) and normal liver tissue samples (15 cases) were collected from the Chinese PLA General Hospital in Beijing between 1 July 2010 and 1 January 2014. The median age of the cancer patients was 55 years old (range 29–71), and the ratio of males/females was 6.3:1. All cancer samples were classified according to TNM staging (AJCC 2010). Forty cases of available matched cancer adjacent tissue paraffin samples were included in this study. All samples were collected following the guidelines approved by the Institutional Review Boards of the Chinese PLA General Hospital with written informed consent from patients. HCC cell lines, which included SNU182, SNU387, HBXF344, SNU475, HepG2, PLC/PRF/5, Huh7, BEL7402, LM3, SNU449, SMMC7721, QGY7703, and Huh1 were previously established from human primary HCC [18]. All cells were maintained in 90% RPMI 1640 (Invitrogen, Carlsbad, CA) supplemented with 10% fetal bovine serum.

### 5-Aza-2′-deoxycytidine treatment

HCC cell lines were split to a low density (30% confluence) 12 h before treatment. Cells were treated with 5-aza-2′-deoxycytidine (5-aza) (Sigma, St. Louis, MO) at a concentration of 2 μM. Growth medium conditioned with 5-aza at a concentration of 2 μM was exchanged every 24 h for a total of 96 h of treatment.

### RNA isolation and semi-quantitative RT-PCR

Total RNA was isolated by Trizol reagent (Life Technologies, Gaithersburg, MD). First-strand cDNA was synthesized according to the manufacturer’s instructions (Invitrogen, Carlsbad, CA). PCR primers for HOXD10 are listed in Additional file 1: Table S1. The primer sets for HOXD10 were designed to span intronic sequences between adjacent exons in order to control for genomic DNA contamination. RT-PCR was amplified for 33 cycles. GAPDH was amplified for 25 cycles as an internal control.

### Bisulfite modification, methylation-specific PCR (MSP), and bisulfite sequencing

DNA was prepared by the proteinase K method. Bisulfite treatment was carried out as previously described [19]. MSP primers were designed according to genomic sequences around transcription start sites (TSS) and synthesized to detect unmethylated (U) and methylated (M) alleles. Bisulfite sequencing (BSSQ) was performed as previously described [20]. BSSQ products were amplified by primers flanking the targeted regions including MSP products. All primers are listed in Additional file 1: Table S1.

### Immunohistochemistry

Immunohistochemistry (IHC) was performed in HCC tissue samples and paired adjacent tissue samples. The HOXD10 antibody was diluted to 1:100 (Abcam, Cambridge, UK). The staining intensity and extent of the staining area were scored using the German semi-quantitative scoring system as previously described [20, 21].

### Construction of HOXD10 expression vector and transfection assay

Full-length *HOXD10* cDNA (GenBank accession number NM_002148.3) was cloned into the pcDNA3.1 expression vector. Transient transfection was performed using Lipofectamine 3000 (Intrivogen, Carlsbad, CA) according to the manufacturer’s instructions.

### Cell viability detection

Cells were plated into 96-well plates at a density of 2 × 10^3^ cells/well, and cell viability was measured by the 3-(4,5-dimethylthiazol-2-yl)-2,5-diphenyltetrazolium bromide (MTT) assay (KeyGEN Biotech, Nanjing, China) at 0, 24, 48, and 72 h. Absorbance was measured using a microplate reader (Thermo Multiskan MK3, MA, USA) at a wavelength of 490 nm.

### Colony formation assay

Cells were seeded into 6-well culture plates at a density of 800 cells per well in triplicate and cultured for 2 weeks. For Huh7 and SMMC7721 cells, growth medium was conditioned with G418 (Invitrogen, Carlsbad, CA) at 300 and 50 μg/ml, respectively, and was exchanged every 24 h. Cells were then fixed with 75% ethanol for 30 min, stained with 0.2% crystal violet (Beyotime, Nanjing, China) for 20 min and counted.

### Flow cytometry

For cell cycle analysis, cells were serum starved 12 h for synchronization and then re-stimulated with 10% FBS for 24 h. Cells were fixed with 70% ethanol and prepared for cell cycle detection using the Cell Cycle Detection Kit (KeyGen Biotech, Nanjing, China). Cells were then sorted by a FACSCalibur (BD Biosciences, San Jose, CA) and analyzed by the Modfit software (Verity Software House, ME, USA). For apoptosis analysis, the Annexin V-FITC/PI Apoptosis Detection Kit (KeyGen Biotechnology, China) was used according to manufacturer’s instructions. Each sample was analyzed by flow cytometry with a FACScan Flow Cytometer (Becton-Dickinson Biosciences, Mansfield, MA).

### Transwell assay

Cells were suspended in serum-free medium. Cells (2 × 10^4^) were placed into the upper chamber of an 8-μm pore size transwell apparatus (Corning, NY, USA) and incubated for 24 h. Cells that migrated to the lower surface of the membrane were stained with crystal violet and counted in three independent high-power fields (×200). For invasion analysis, cells (3 × 10^4^) were seeded into the upper chamber of a transwell apparatus coated with Matrigel (BD Biosciences, San Jose, CA) and incubated for 48 h. Cells that invaded into the lower membrane surface were stained with crystal violet and counted in three independent high-power fields (×200).

### Chromatin immunoprecipitation

Chromatin immunoprecipitation (ChIP) was performed in HOXD10 highly expressed Huh1 cells using HOXD10 monoclonal antibody (Life Span Bio Sciences, Inc., WA, USA) or normal rabbit IgG (negative control) according to the EpiTect ChIP One Day Kit (Qiagen, Hilden, Germany). Two primers encompassing HOXD10 binding sites in different regions of the *IGFBP3* promoter were designed as shown in Additional file 1: Table S1.

### SiRNA knockdown technique

SiRNAs targeting HOXD10 and the RNAi negative control duplex were used in this study. The sequences of the siRNAs are listed in Additional file 1: Table S1 (Gene Pharma Co, Shanghai, China). The RNAi oligonucleotide and RNAi negative control duplex were transfected into HOXD10 highly expressing QGY7703 and Huh1 cells.

### Western blot

Proteins from HCC cells were collected 48 h after transfection. For extracellular signal-regulated kinase (ERK) signaling analysis, cells were starved with serum-free medium for 24 h after transfection. These cells were then stimulated with medium containing 10% serum for 15 to 60 min before collection. Western blot was performed as described previously [21]. Antibodies were diluted according to manufacturer’s instructions. The primary antibodies were as follows: HOXD10 (Life Span Bio Sciences, Inc., WA, USA), IGFBP3 (Protein Tech Group, Chicago, IL, USA), ERK1/2 (Bioworld Tech, MN, USA), p-ERK1/2 (Bioworld Tech, MN, USA), MMP2 (Protein Tech Group, Chicago, IL, USA), MMP9 (Protein Tech Group, Chicago, IL, USA), cyclinB1 (Protein Tech Group, Chicago, IL, USA), cdc-2 (Protein Tech Group, Chicago, IL, USA), bcl-2 (Protein Tech Group, Chicago, IL, USA), cleaved caspase 3 (Protein Tech Group, Chicago, IL, USA), and β-actin (Bioworld Tech, MN, USA).

### HOXD10 unexpressed and re-expressed SMMC7721 cell xenograft mouse model

Stably transfected SMMC7721 cell line with pLenti6 vector or pLenti6-HOXD10 vector (6 × 10^6^ cells diluted in phosphate-buffered saline and matrigel mixed at the ratio of 1:1) were injected subcutaneously into the dorsal right side of 4-week-old female Balb/c nude mice. Each group includes six mice. Tumor volume was measured every 3 days. Tumor volume was calculated according to the formula: *V* = *L* × *W*2/2, where *V* represents volume (mm3), *L* represents biggest diameter (mm), and *W* represents smallest diameter (mm). Mice were sacrificed on the 39th day after inoculation and tumor was weighted. All procedures were approved by the Animal Ethics Committee of the Chinese PLA General Hospital.

### Statistical analysis

SPSS 17.0 software (IBM, NY, USA) was used for data analysis. Categorical variables were analyzed using the chi-squared test or Fisher’s exact test. The two-tailed independent sample *t* test was applied to determine the statistical significance of the differences between the two experimental groups. For matched HCC and adjacent tissue samples, paired Student’s *t* test was employed. Survival rates were calculated by the Kaplan–Meier method, and differences in survival curves were evaluated using the log-rank test. Cox proportional hazards models were fit to determine independent associations of HOXD10 methylation with 3-year OS outcomes. Two-sided tests were used to determine the significance, and *P* < 0.05 was considered as statistically significant.

## Results

### HOXD10 is silenced by promoter region hypermethylation in HCC cells

**Fig. 1:**
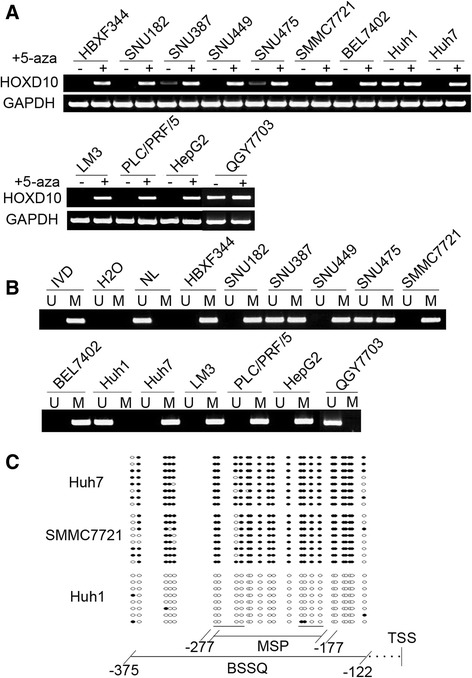
*HOXD10* expression and methylation status in human HCC cells. **a** Semi-quantitative RT-PCR shows HOXD10 expression levels in HCC cell lines. HBXF344, SNU182, SNU387, SNU449, SNU475, SMMC7721, BEL7402, Huh1, Huh7, LM3, PLC/PRF/5, HepG2, and QGY7703 are HCC cell lines. 5-AZA: 5-aza-2′-deoxycytidine; GAPDH: internal control of RT-PCR; (−): absence of 5-AZA; (+): presence of 5-AZA. **b** MSP results of *HOXD10* in HCC cell lines. U: unmethylated alleles; M: methylated alleles; IVD: in vitro methylated DNA, serves as methylation control; NL: normal peripheral lymphocytes DNA, serves as unmethylated control; H_2_O: double distilled water. **c** BSSQ results of *HOXD10* in SMMC7721, Huh7 and Huh1 cells. Double-headed arrow: MSP PCR product size was 100 bp and bisulfite sequencing focused on a 254 bp region of the CpG island (from − 375 to − 122) around the *HOXD10* transcription start site. Filled circles: methylated CpG sites, open circles: unmethylated CpG sites. TSS: transcription start site

Semi-quantitative RT-PCR was employed to detect the expression of HOXD10 in HCC cells. Loss of HOXD10 expression was found in SNU182, SNU449, HBXF344, SMMC7721, Huh7, HepG2, LM3, PLC/PRF/5, and BEL7402 cells. Reduced expression of HOXD10 was found in SNU387 and SNU475 cells. HOXD10 was highly expressed in QGY7703 and Huh1 cells (Fig. 1a). MSP was employed to detect promoter region methylation. MSP primers were designed around the transcription start site in the CpG islands within the *HOXD10* gene promoter region. Complete methylation was found in SNU182, SNU449, HBXF344, SMMC7721, Huh7, HepG2, LM3, PLC/PRF/5, and BEL7402 cells, partial methylation was found in SNU387 and SNU475 cells, and unmethylation was found in QGY7703 and Huh1 cells (Fig. 1b). These results demonstrate that loss or reduced expression of HOXD10 is correlated with promoter region methylation.

To further validate that the expression of HOXD10 was regulated by promoter region methylation, HCC cells were treated with the DNA methylation transferase inhibitor, 5-aza-2′-deoxycytidine (5-aza). Re-expression of HOXD10 was found in SNU182, SNU449, HBXF344, SMMC7721, Huh7, HepG2, LM3, PLC/PRF/5, and BEL7402 cells, and increased expression of HOXD10 was found in SNU387 and SNU475 cells. No expression change was found in QGY7703 and Huh1 cells (Fig. 1a). These results suggest that HOXD10 expression is regulated by promoter region methylation in HCC cells. To further validate the MSP results and examine the methylation density in the promoter region, BSSQ was performed in SMMC7721, Huh7, and Huh1 cells. BSSQ results were consistent with the MSP results, showing dense promoter region methylation in SMMC7721 and Huh7 cells and no methylation in Huh1 cells (Fig. 1c).

### HOXD10 is frequently methylated in human primary HCC, and reduced expression of HOXD10 is associated with promoter region hypermethylation

**Fig. 2:**
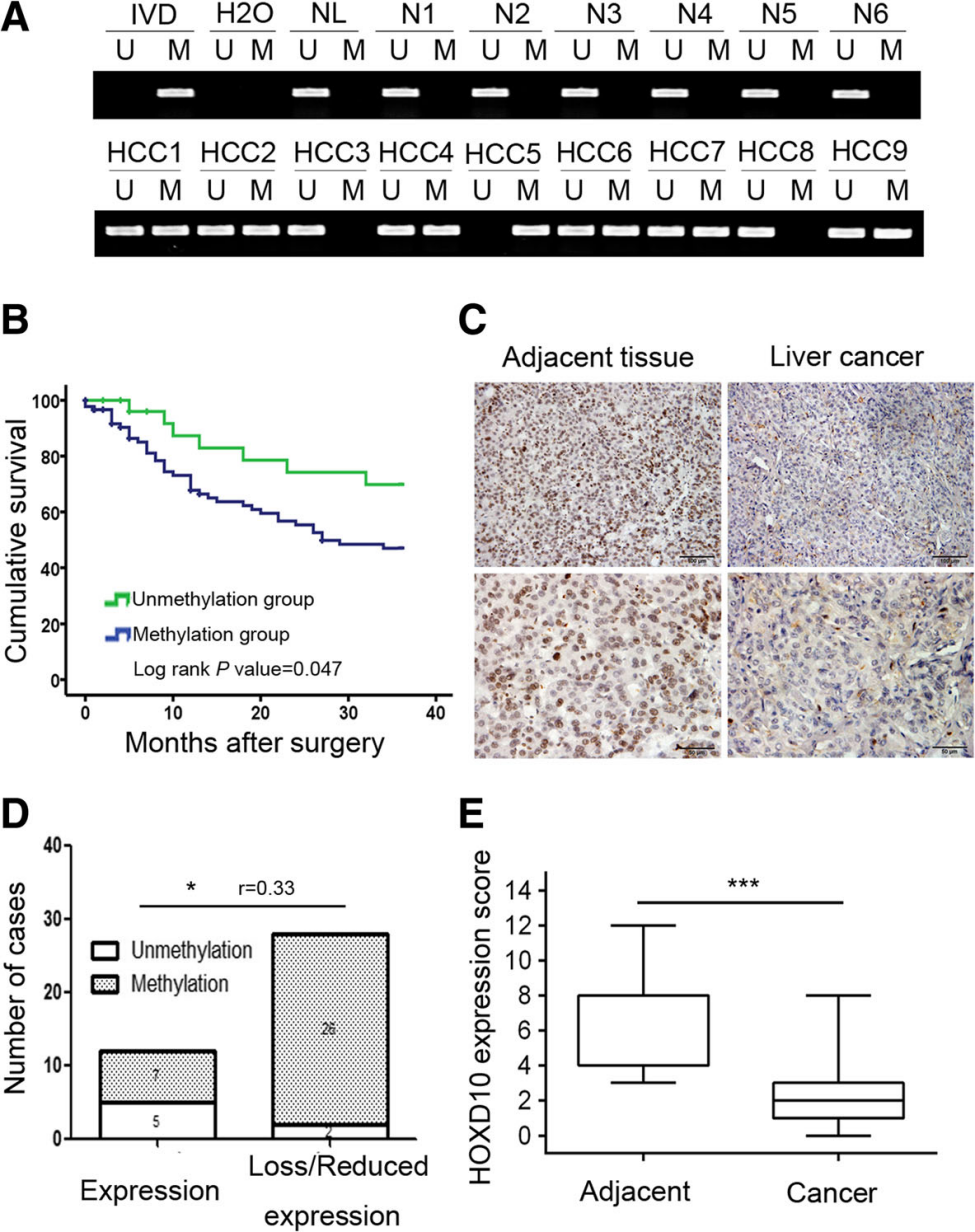
The expression and methylation status of *HOXD10* in primary HCC. **a** Representative MSP results of *HOXD10* in normal liver tissue samples and primary HCC samples. N: normal liver tissue samples; HCC: primary HCC samples. **b** The 3-year overall survival curves for patients in the methylated and unmethylated groups (*P* < 0.05). **c** Representative IHC results show HOXD10 expression in HCC tissue and adjacent tissue samples (top: ×200; bottom: ×400). **d** The expression of HOXD10 and DNA methylation status is shown as a bar diagram. Reduced expression of HOXD10 was significantly associated with promoter region hypermethylation. *P < 0.05, *r* = 0.33. **e** HOXD10 expression scores are shown as box plots; horizontal lines represent the median score; the bottom and top of the boxes represent the 25th and 75th percentiles, respectively; vertical bars represent the range of data. Expression of HOXD10 was significantly different between adjacent tissue and HCC tissue in 40-matched HCC samples. ***P < 0.001

To determine the methylation status of *HOXD10* in human primary HCC, 117 cases of primary HCC and 15 cases of normal liver tissue samples were examined by MSP. *HOXD10* was methylated in 76.9% (90/117) of primary HCC samples, but no methylation was detected in normal liver tissue samples (Fig. 2a). As shown in Table 1, methylation of HOXD10 was more frequently in patients with vessel cancerous embolus (*p* < 0.05) and poorly differentiated tumors (*P* < 0.05), but no association was found between HOXD10 methylation and age, gender, HBV infection, cirrhosis, tumor size, number of lesions, TNM stage, and lymph node metastasis (all *P* > 0.05).The median follow-up period for patients was 23 months (range, 0–76 months).Four patients in the unmethylated group and 17 patients in the methylated group were lost to follow-up. Kaplan–Meier plots indicated that methylation of HOXD10 was associated with poor 3-year overall survival (OS) (*P* = 0.048, Fig. 2b). While, according to Cox proportional hazards model analysis, HOXD10 methylation was not an independent prognostic factor for 3-year OS after adjusting for tumor differentiation, vessel cancerous embolus, and TNM stage (*P* = 0.127, Table 2).

**Table 1:** Clinical factors and *HOXD10* methylation in 117 cases of HCC samples

Clinical factor	No.	*HOXD10* methylation status		*P value
		Unmethylated *n* = 27 (23.1%)	Methylated *n* = 90 (76.9%)	
Age (year)				0.799
< 60	80	19	61	
≥ 60	37	8	29	
Gender				0.248
Male	101	21	80	
Female	16	6	10	
HBV infection				0.762
Yes	85	19	66	
No	32	8	24	
Liver cirrhosis				0.093
Yes	88	17	71	
No	29	10	19	
Tumor size (cm)				0.231
≤ 5	37	6	31	
> 5	80	21	59	
Number of lesions				0.681
1	92	22	70	
≥ 1	25	5	20	
Differentiation				0.044*
Well	8	4	4	
Moderate	74	19	55	
Poor	35	4	31	
TNM stage				0.377
Stage I + stage II	52	14	38	
Stage III + stage IV	65	13	52	
Lymph node metastasis				0.867
Negative	112	26	86	
Positive	5	1	4	
Vessel cancerous embolus				0.049*
Negative	87	24	63	
Positive	30	3	27	

*P values are obtained from chi-square test, significant difference, *P < 0.05

**Table 2:** Univariate and multivariate analysis of HOXD10 methylation status with 3-year overall survival (OS) in HCC patients

Clinical factor	3-year OS			
	Univariate analysis		Multivariate analysis	
	HR (95% CI)	*P* value	HR (95% CI)	*P* value
Age (< 60 vs. ≥ 60 years)	1.306 (0.775–2.201)	0.317		
Gender (male vs. female)	0.717 (0.376–1.368)	0.312		
HOXD10 (methylation vs. unmethylation)	1.920 (1.006–3.664)	0.048*	1.676 (0.864–3.252)	0.127
HBV infection (yes vs. no)	0.664 (0.374–1.178)	0.161		
Liver cirrhosis (yes vs. no)	0.888 (0.513–1.538)	0.672		
Tumor size (≤ 5 vs. > 5 cm)	0.707 (0.412–1.212)	0.207		
Number of lesions (1 vs. ≥ 1)	0.770 (0.439–1.349)	0.361		
Differentiation (well or moderate vs. poor)	0.787 (0.476–1.301)	0.351	1.267 (0.740–2.172)	0.388
TNM stage (stage I + stage II vs. stage III + stage IV)	0.391 (0.233–0.656)	0.000***	0.502 (0.279–0.902)	0.021*
Lymph node metastasis (negative vs. positive)	0.431 (0.172–1.075)	0.071		
Vessel cancerous embolus (negative vs. positive)	0.356 (0.217–0.584)	0.000***	0.503 (0.287–0.884)	0.017*

*P < 0.05, ***P < 0.001

The expression of HOXD10 was evaluated by immunohistochemistry (IHC) in 40 cases of available matched primary HCC and adjacent tissue samples. Staining of HOXD10 was mainly localized in the nucleus, and its expression was significantly reduced in primary HCC compared to adjacent tissue samples (*P* < 0.001, Fig. 2c, e). In 40 cases of available primary HCC, loss or reduced expression of HOXD10 was found in 28 cases. Of these 28 case samples, 26 cases were methylated and 2 cases were unmethylated. Loss or reduced expression of HOXD10 was significantly associated with promoter region hypermethylation (*P* < 0.05, *r* = 0.33, Fig. 2d). These results indicate that HOXD10 expression is regulated by promoter region methylation in primary HCC.

### HOXD10 suppresses proliferation of HCC cells

**Fig. 3:**
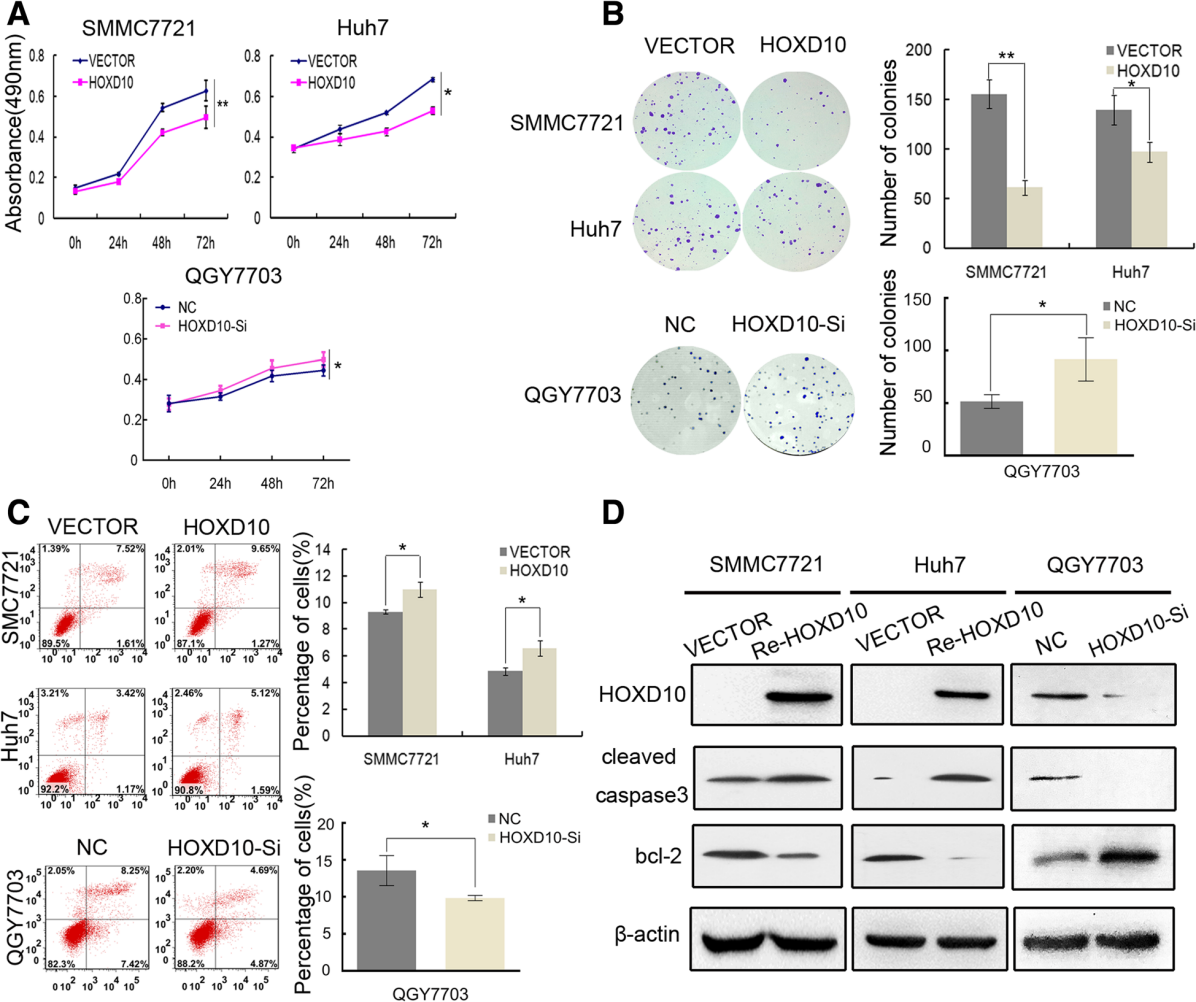
The effect of HOXD10 on HCC cell proliferation and apoptosis. **a** Growth curves represent the cell viability analyzed by the MTT assay in HOXD10 re-expressed and unexpressed SMMC7721 and Huh7 cells, as well as in QGY7703 cells before and after knockdown of HOXD10. Each experiment was repeated in triplicate. *P < 0.05, **P < 0.01. **b** Colony formation results show that colony number was reduced by re-expression of HOXD10 in SMMC7721 and Huh7 cells, while increased by knockdown of HOXD10 in QGY7703 cells. Each experiment was repeated in triplicate. Average number of tumor clones is represented by bar diagram. *P < 0.05, **P < 0.01. **c** Flow cytometry results show induction of apoptosis by re-expression of HOXD10 in SMMC7721 and Huh7 cells, while reduction of apoptosis was found after knockdown of HOXD10 in QGY7703 cell. * *P* < 0.05. **d** Western blots show the effects of HOXD10 on the levels of cleaved caspase 3 and bcl-2 expression in SMMC7721, Huh7, and QGY7703 cells. VECTOR: control vector, Re-HOXD10: HOXD10 expressing vector, β-actin: internal control

To evaluate the effects of HOXD10 on HCC cell proliferation, cell viability was determined by the MTT assay. The OD value was 0.63 ± 0.05 vs. 0.50 ± 0.05 (*P* < 0.01) and 0.68 ± 0.01 vs. 0.53 ± 0.02 (*P* < 0.05) before and after restoration of HOXD10 expression in HOXD10 unexpressed SMMC7721 and Huh7 cells, respectively. The OD value was reduced significantly (Fig. 3a). In HOXD10 highly expressed QGY7703 cells, the OD value was 0.45 ± 0.03 vs. 0.50 ± 0.04 before and after knockdown of HOXD10 expression. The OD value was increased significantly (*P* < 0.05, Fig. 3a). These results suggest that HOXD10 suppresses HCC cell viability. Colony formation assays were performed to evaluate the effect of HOXD10 on clonogenicity. As shown in Fig. 3b, the colony number was 155.67 ± 14.64 vs. 61.0 ± 7.55 in SMMC7721 cells (*P* < 0.01) and 139.67 ± 15.18 vs. 97.33 ± 10.07 in Huh7 cells (*P* < 0.05) before and after re-expression of HOXD10, showing significant reduction in colony formation with HOXD10 re-expression. In HOXD10 highly expressed QGY7703 cells, the colony number was 51.67 ± 6.43 vs. 91.67 ± 20.50 before and knockdown of HOXD10. The colony number was significantly increased after knockdown of HOXD10 expression (*P* < 0.05, Fig. 3b). These results demonstrate that HOXD10 suppresses HCC cell growth.

### HOXD10 induces cell apoptosis

To explore the role of HOXD10 in apoptosis, flow cytometry was performed. The percentages of apoptotic cells were 9.30 ± 0.15% vs. 11.00 ± 0.57% in SMMC7721 cells (*P* < 0.05) and 4.86 ± 0.26% vs. 6.57 ± 0.55% in Huh7 cells (*P* < 0.05) before and after re-expression of HOXD10. The percentage of apoptotic cells was increased significantly (Fig. 3c) after re-expression of HOXD10. In HOXD10 highly expressed QGY7703 cells, the percentage of apoptotic cells was 13.56 ± 2.03% before knockdown of HOXD10 and 9.83 ± 0.36% after knockdown of HOXD10. The percentage of apoptotic cells was reduced significantly (*P* < 0.05, Fig. 3c). To further validate HOXD10-induced apoptosis in HCC cells, cleaved capase-3 and bcl-2 levels were examined by western blot. The levels of cleaved capase-3 were increased and the levels of bcl-2 were reduced after re-expression of HOXD10 in SMMC7721 and Huh7 cell. In HOXD10 highly expressed QGY7703 cells, the levels of cleaved capase-3 were reduced and the levels of bcl-2 were increased after knockdown of HOXD10 (Fig. 3d). These results suggest that HOXD10 induces apoptosis in HCC cells.

### HOXD10 induces G2/M phase arrest

**Fig. 4:**
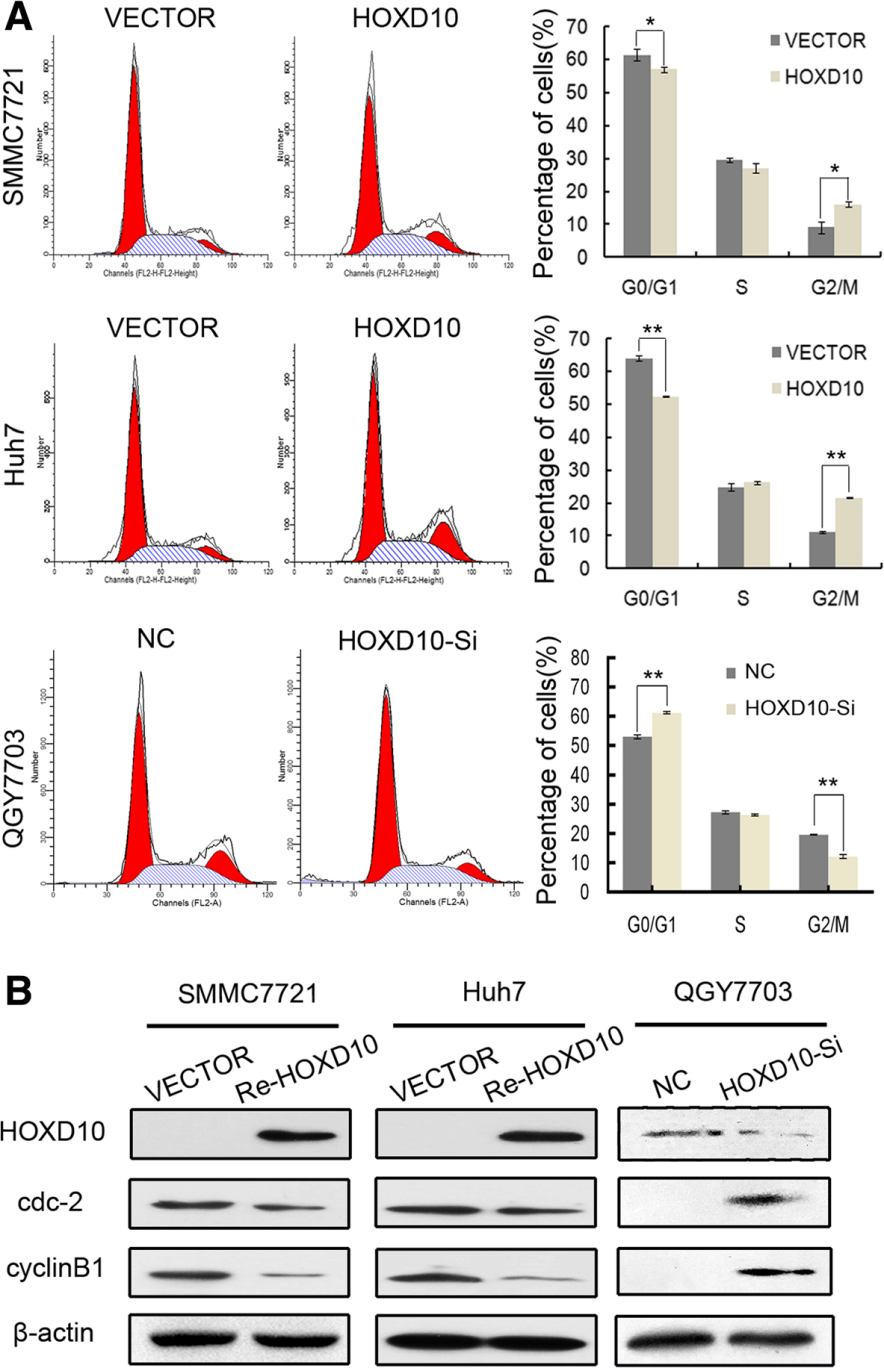
The effect of HOXD10 on HCC cell cycle. **a** Cell phase distribution in HOXD10 unexpressed and re-expressed SMMC7721 and Huh7 cells, as well as in QGY7703 cells before and after knockdown of HOXD10. The ratio is presented by bar diagram. Each experiment was repeated three times. *P < 0.05, **P < 0.01. **b** The expression of HOXD10, cyclinB1, and cdc-2 was detected by western blot in HOXD10 unexpressed and re-expressed SMMC7721 and Huh7 cells, as well as in QGY7703 cells before and after knockdown of HOXD10. β-actin: internal control

The role of HOXD10 in the cell cycle was analyzed by flow cytometry. As shown in Fig. 4a, the distribution of cell phase in HOXD10 unexpressed and re-expressed SMMC7721 cells was 61.35 ± 1.75% vs. 56.86 ± 0.69% in G0/G1 phase (*P* < 0.05), 29.55 ± 0.59% vs. 27.04 ± 1.43% in S phase, and 9.10 ± 1.76% vs. 16.09 ± 0.80% in G2/M phase (*P* < 0.05). In Huh7 cells, the cell phase distribution was 63.95 ± 0.92% vs. 52.27 ± 0.23% in G0/G1 phase (*P* < 0.01), 24.85 ± 1.09% vs. 26.16 ± 0.38% in S phase, and 11.20 ± 0.36% vs. 21.58 ± 0.15% in G2/M phase (*P* < 0.01) before and after restoration of HOXD10 expression (Fig. 4a). G2/M phase was increased significantly after re-expression of HOXD10 in HCC cells. To further validate these results, siRNA knockdown technique was employed. The cell phase distribution was 53.00 ± 0.61% vs. 61.28 ± 0.38% in G0/G1 phase (*P* < 0.01), 27.30 ± 0.52% vs. 26.40 ± 0.40% in S phase, and 19.67 ± 0.12% vs. 12.20 ± 0.61% in G2/M phase before and after knockdown of HOXD10 in HOXD10 highly expressed QGY7703 cells (Fig. 4a). The percentage of G2/M phase was reduced significantly after knockdown of HOXD10 (*P* < 0.01). These results suggest that HOXD10 induces G2/M phase arrest in HCC cells.

The induction of G2/M checkpoint arrest by HOXD10 was further validated by detecting G2/M phase-related proteins. The expression levels of cyclinB1 and cdc-2 were dramatically reduced after re-expression of HOXD10 in SMMC7721 and Huh7 cells, and the levels of cyclinB1 and cdc-2 expression were increased obviously after knockdown of HOXD10 in HOXD10 highly expressed QGY7703 cells (Fig. 4b). Above results suggest that HOXD10 inhibits cell proliferation in HCC.

### HOXD10 suppresses cell invasion and migration in HCC

**Fig. 5:**
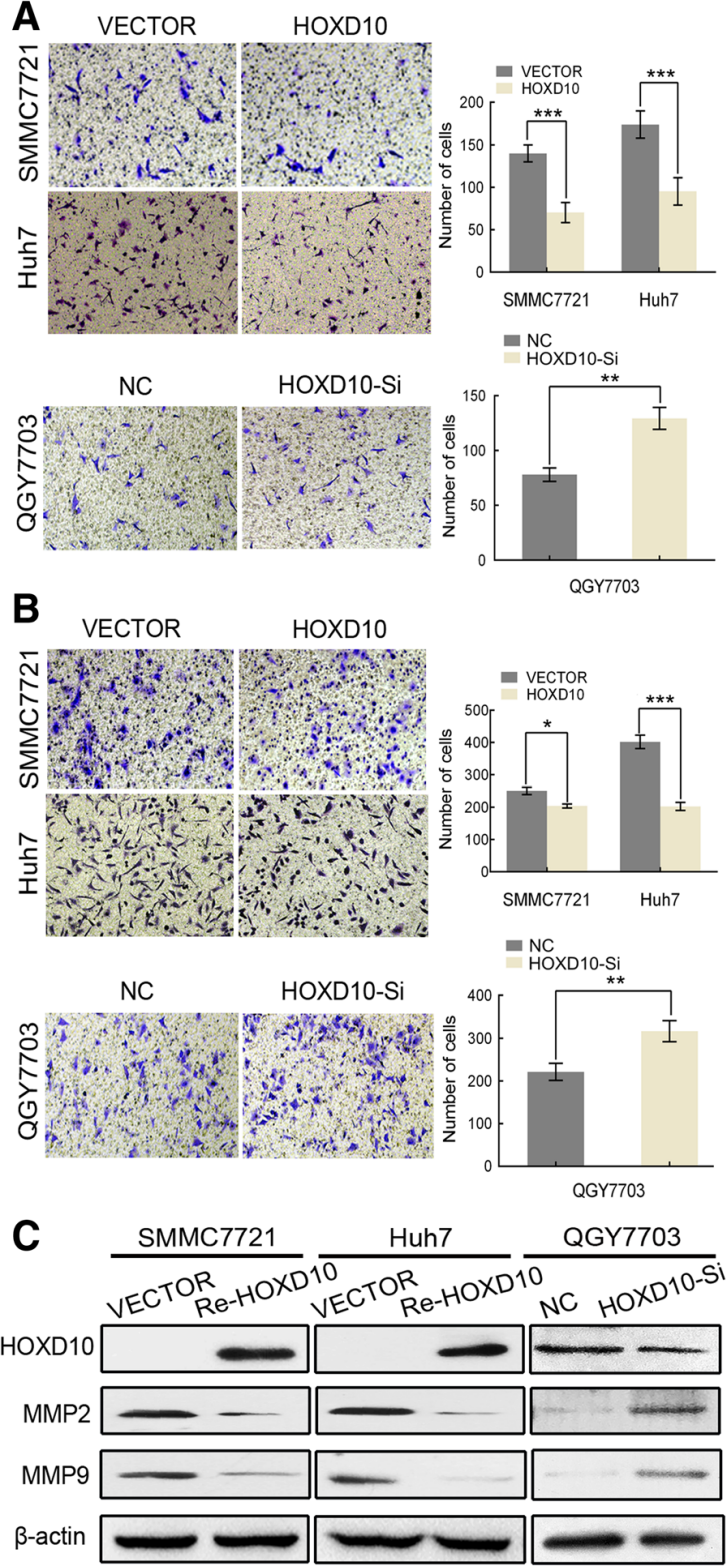
HOXD10 inhibits cell invasion and migration in HCC cells. **a** Cell invasion in HOXD10 unexpressed and expressed SMMC7721 and Huh7 cells, as well as in QGY7703 cells before and after knockdown of HOXD10. The number of cell invading to the lower chamber is presented by bar diagram. Each experiment was repeated three times. **P < 0.01, ***P < 0.001. **b** Cell migration in HOXD10 unexpressed and expressed SMMC7721 and Huh7 cells, as well as in QGY7703 cells before and after knockdown of HOXD10. The migratory cell number is presented by bar diagram. Each experiment was repeated three times. *P < 0.05, **P < 0.01, ***P < 0.001. **c** The expression levels of HOXD10, MMP2, and MMP9 were detected by western blot

The transwell assay was employed to evaluate the effects of HOXD10 on cell invasion. The number of cells for each high-power field under the microscope was 140.00 ± 10.00 vs. 70.33 ± 11.68 in SMMC7721 cells and 159.00 ± 14.73 vs. 93.33 ± 7.51 in Huh7 cells before and after restoration of HOXD10 expression. The number of invasive cells was reduced significantly after re-expression of HOXD10 in SMMC7721 and Huh7 cells (all *P* < 0.001, Fig. 5a). In HOXD10 highly expressed QGY7703 cells, the number of invasive cells for each high-power field under the microscope was 78.00 ± 6.08 vs 129.30 ± 10.07 before and after knockdown of HOXD10. The number of invasive cells was increased significantly (*P* < 0.01, Fig. 5a). To explore the mechanism of HOXD10 in HCC cell invasion, MMP2 and MMP9 were examined by western blot. The expression levels of MMP2 and MMP9 were reduced after re-expression of HOXD10 in SMMC7721 and Huh7 cells. While, the expression levels of MMP2 and MMP9 was increased obviously after knockdown of HOXD10 in HOXD10 highly expressed QGY7703 cells (Fig. 5c). The results suggest that HOXD10 inhibits cell invasion in HCC cells.

Next, the transwell assay was performed in the absence of extracellular matrix (ECM) gel coating to explore the effects of HOXD10 on cell migration. The numbers of migrated cells for each high-power field under the microscope were 251.00 ± 11.36 vs. 197.67 ± 11.02 in SMMC7721 cells (*P* < 0.05) and 402.00 ± 20.52 vs. 203.00 ± 12.29 (*P* < 0.001) in Huh7 cells before and after restoration of HOXD10 expression. The number of migrated cells was 221.70 ± 19.76 vs. 317.00 ± 24.64 before and after knockdown of HOXD10 in HOXD10 highly expressed QGY7703 cells (*P* < 0.01, Fig. 5b). The results indicate that HOXD10 inhibits cell migration in HCC cells.

### HOXD10 inhibits ERK signaling in HCC cells

**Fig. 6:**
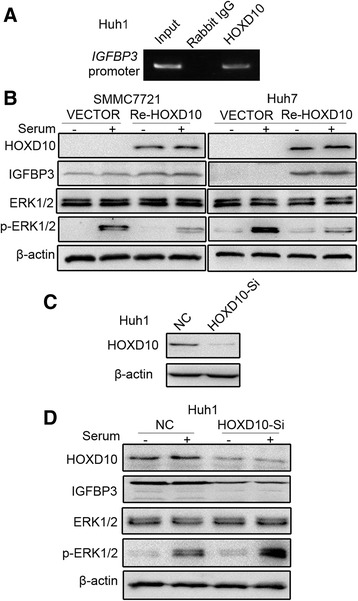
HOXD10 interacts with IGFBP3 and inhibits ERK1/2 phosphorylation in HCC. **a** ChIP results show that HOXD10 binds to the promoter region of *IGFBP3*. **b** Western blots show the levels of HOXD10, IGFBP3, ERK1/2 and p-ERK1/2 in SMMC7721 and Huh7 cells. β-actin: internal control. −: no serum stimulation. +: serum stimulation. **c** The expression levels of HOXD10 after knockdown by siRNA in Huh1 cells. HOXD10-Si: siRNA, NC: negative control. **d** The levels of HOXD10, IGFBP3, ERK1/2, and p-ERK1/2 were detected by western blot after knockdown of HOXD10 in Huh1 cells. β-actin: internal control. −: no serum stimulation. +: serum stimulation

HOXD10 has been demonstrated to act as a transcription factor targeting the promoter region of IGFBP3 in gastric cancer [15, 22]. IGFBP3 may activate different signaling pathways in different cancers [23]. In the NSCLC cell line H1299, IGFBP3 interacts with and inactivates ERK1/2 by inhibiting ERK1/2 phosphorylation [24]. In human HCC, the signaling pathway involving HOXD10 remains unclear. To further understand the mechanism of HOXD10 in HCC and determine whether it binds to IGFBP3, ChIP assays were performed. The *IGFBP3* promoter region was pulled down by the HOXD10 antibody in HOXD10 highly expressing Huh1 cells (Fig. 6a). Results of the ChIP assay suggest that HOXD10 interacts with the promoter region of *IGFBP3*. To further analyze the role of HOXD10, the expression levels of IGFBP3 were examined by western blot in HOXD10 unexpressed and re-expressed SMMC7721 and Huh7 cells. The expression of IGFBP3 increased after re-expression of HOXD10, suggesting that HOXD10 upregulates IGFBP3 in HCC cells (Fig. 6b).

Next, we detected phosphorylation status of ERK1/2 by western blot to further explore the mechanism of IGFBP3 in regulation of downstream signaling. After 24 h of serum starvation followed by 10% serum stimulation for 45 min, the levels of phosphorylated ERK1/2 were reduced in HOXD10 re-expressed SMMC7721 cells compared to HOXD10 unexpressed SMMC7721 cells. Similarly, the levels of phosphorylated ERK1/2 were reduced by HOXD10 in Huh7 cells after 10% serum stimulation for 30 min. These results indicate that HOXD10 inhibits ERK1/2 phosphorylation by upregulating IGFBP3 expression (Fig. 6b). To further validate the inhibitory effect of HOXD10 on ERK signaling, siRNA knockdown technique was used in HOXD10 highly expressing Huh1 cells. HOXD10-SiR1 effectively knocked down HOXD10 in Huh1 cells (Fig. 6c). The levels of phosphorylated ERK1/2 were increased by knockdown of HOXD10 in 10% FBS-stimulated Huh1 cells (Fig. 6d). These results suggest that HOXD10 inhibits ERK signaling by upregulating IGFBP3 in HCC.

### HOXD10 suppresses human HCC cell tumor growth in xenograft mice

**Fig. 7:**
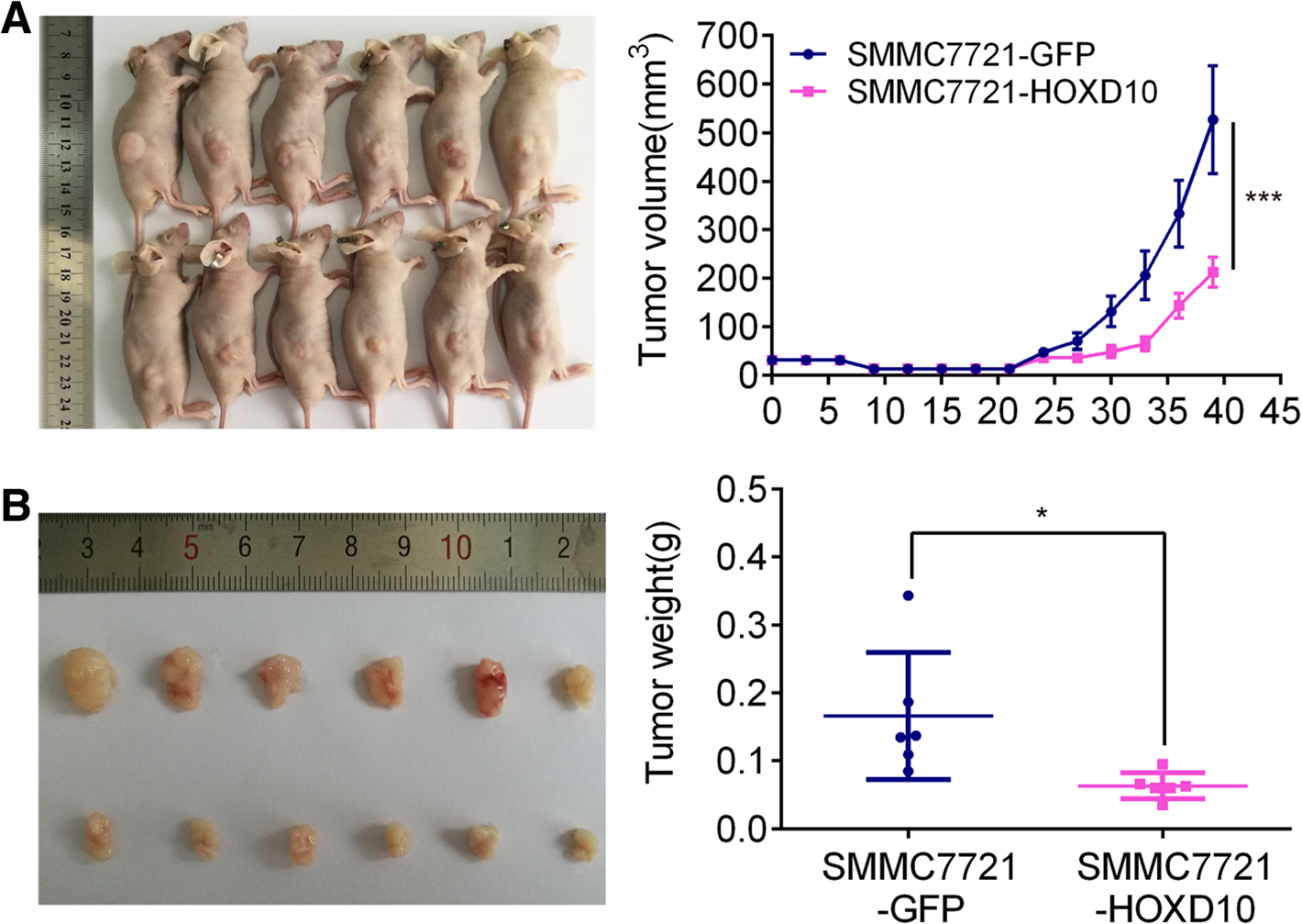
HOXD10 suppresses human HCC cell tumor growth in xenograft mice. **a** Tumor growth curve for xenograft mice subcutaneously burdened with SMMC7721 cells stable transfected with HOXD10 or GFP control. ***P < 0.001. **b** Tumor weights of nude mice 39 days after inoculation with SMMC7721 cells in which HOXD10 is re-expressed or unexpressed. *P < 0.05

To further investigate the role of HOXD10 in human HCC, a xenograft mouse model was employed. HOXD10 unexpressed and re-expressed SMMC7721 cells were inoculated into nude mice subcutaneously. The tumor volume in HOXD10 unexpressed and re-expressed SMMC7721 cell transplanted xenograft mice was 527.22 ± 271.23 mm^3^ vs. 212.00 ± 75.93 mm^3^. The tumor volume was smaller in HOXD10 re-expressed SMMC7721 cell xenograft mice compared to HOXD10 unexpressed SMMC7721 cell xenograft mice (*P* < 0.001, Fig. 7a). The tumor weights were 0.17 ± 0.09 g vs. 0.06 ± 0.02 g (*P* < 0.05) in HOXD10 unexpressed and re-expressed SMMC7721 cell xenografts. The tumor weight was significantly reduced after re-expression of HOXD10 (*P* < 0.05, Fig. 7b). The results demonstrate that HOXD10 suppresses HCC cell growth in vivo.

## Discussion

HCC is a complex disease. Hepatocarcinogenesis involves hepatocyte injury, inflammation, proliferation, and genomic instability, which lead to alteration in several oncogenic pathways, including EGFR, AKT, WNT, and ERK signaling [25–28]. Four clusters and 39 Hox genes have been identified in humans. Hox genes are conserved across species and encode transcription factors that are defined by the DNA-binding domain called the homeodomain. Hox proteins can function as monomers or homodimers to directly drive the transcription of downstream targets. The effects exerted by Hox genes are varied in different pathways with notable tissue specificity. Hox genes may serve as oncogenes or tumor suppressor genes in different cancer types [10]. The expression of HOXD10 was lost during the malignant progression of breast cancer, while the expression of HOXD10 was increased in human head and neck cancer [29, 30]. The expression and regulation of HOXD10 in human HCC remains unclear.

In this study, we demonstrated that the expression of HOXD10 is reduced/lost frequently in HCC, and the expression of HOXD10 is regulated by promoter region methylation. HOXD10 methylation was associated with vessel cancerous embolus, tumor cell differentiation, and the 3-year survival rate. The results suggest that HOXD10 methylation may serve as a poor prognostic marker of HCC. Following up of this cohort, we only obtained 3-year OS data. Analyzing by Cox proportional hazards model, HOXD10 methylation was not an independent prognostic marker for 3-year OS after adjusting for tumor differentiation, vessel cancerous embolus, and TNM stage. Increasing the cohort number is necessary in our future study. To further clarify the function of HOXD10 in HCC, we analyzed the effects of HOXD10 on cell proliferation, apoptosis, cell cycle, cell invasion, and migration in HCC cells. In our study, HOXD10 suppressed HCC cell proliferation, induced apoptosis, and G2/M phase arrest and inhibited cell invasion and migration. Li et al. found that miR-224 directly targeted HOXD10, which triggered the down-stream p-PAK4/MMP-9 signaling pathway, subsequently contributing to the regulation of cell migration and invasion [31]. Our study found that HOXD10 methylation is associated with vessel cancerous embolus and HOXD10 suppresses HCC cell invasion and migration. These results suggest that HOXD10 is a tumor suppressor in human HCC. The role of HOXD10 in HCC was validated by xenograft mice model in vivo.

HOXD10 targeted the *IGFBP3* gene promoter region and upregulated its expression in gastric cancer [22]. Therefore, we analyzed the expression of IGFBP3 in HCC cells by western blot. The expression of IGFBP3 was upregulated by HOXD10. IGFBP3 was previously reported to interact with and inactivate ERK1/2 by inhibiting ERK1/2 phosphorylation in human non-small cell lung cancer [24]. We analyzed the effects of HOXD10 on the ERK pathway in HCC. The levels of phosphorylated ERK1/2 were reduced by HOXD10. These results were validated using the siRNA knockdown technique.

## Conclusion

HOXD10 is frequently methylated in human HCC, and the expression of HOXD10 is regulated by promoter region methylation. Methylation of HOXD10 was associated with vessel cancerous embolus, tumor cell differentiation, and the 3-year survival rate in human HCC. HOXD10 suppresses HCC cell growth both in vitro and in vivo. HOXD10 suppresses human HCC by inhibiting ERK signaling.

## Additional file

Additional file 1: Table S1. Primer sequences. (DOCX 13 kb)

## Abbreviations

5-aza: 5-Aza-2′-deoxycytidine
bcl-2: B cell lymphoma-2 B
BSSQ: Bisulfite sequencing
*CCND1*: Cyclin D1
cdc-2: Cyclin-dependent kinase 1
ChIP: Chromatin immunoprecipitation
EGFR: Epidermal growth factor receptor
ERK: Extracellular signal-regulated kinase
GADPH: Glyceraldehyde-3-phosphate dehydrogenase
HBV: Hepatitis B virus
HCC: Hepatocellular carcinoma
*HOX*: Homeobox
IGFBP3: Insulin-like growth factor-binding protein 3
IHC: Immunohistochemistry
MMP2: Matrix metalloprotein 2
MMP9: Matrix metalloprotein9
MTT: 3-(4,5-Dimethylthiazol-2-yl)-2,5-diphenyltetrazolium bromide
NSCLC: Nonsmall-cell lung cancer
MSP: Methylation-specific PCR
RT-PCR: Reverse transcriptase polymerase chain reaction
*VEGFA*: Vascular endothelial growth factor A
