# Supplementary material for: Silencing HOXD10 by promoter region hypermethylation activates ERK signaling in hepatocellular carcinoma
Source: Clin Epigenetics. 2017 Oct 23;9:116. doi: 10.1186/s13148-017-0412-9 (PMC5654145; doi:10.1186/s13148-017-0412-9)
Supplement: Additional file 1: Table S1. — Primer sequences. (DOCX 13 kb) [file 13148_2017_412_MOESM1_ESM.docx]

**Table S1. Primers used in this study**

| Primers | Sequence (5’-3’) |
| --- | --- |
| RT-HOXD10-F | GATGAACGAG CCCGTGAGCG |
| RT-HOXD10-R | CTGCCACTCTTTGCAGTG AGC |
| RT-GADPH-F | GACCACAGTCCATGCCATCAC |
| RT-GADPH-R | GTCCACCACCCTGTT GCTGTA |
| MSP-*HOXD10*-MF | CGGTT GGG TTTAGGGGTCGAAGC |
| MSP-*HOXD10*-MR | AACAACCGACC CAACGCTCTCCG |
| MSP-*HOXD10*-UF | GTTGGTTGGGT TAGGGGTTGAAGT |
| MSP-*HOXD10*-UR | AAAACAACCAA CCCAACACTCTCCA |
| BSSQ-*HOXD10*-F | GTTGGCGTTAGGGTA TTTTGG |
| BSSQ-*HOXD10*-R | CACGT ACTCCAAAAACCTAACC |
| CDS-HOXD10-F | AAGCTTATGTCCTTTCCCAACAGCTCTCC |
| CDS-HOXD10-R | CTCGAGCTAAGAAAACGTGAGGTTGGCGG |
| ChIP-HOXD10-A1-F | AAGATTAACTTCACCCAAGGC |
| ChIP-HOXD10-A1-R | AGGTGGATAGGTGACTTG |
| ChIP-HOXD10-A2-F | GCCGACAGGAGTTACAG |
| ChIP-HOXD10-A2-R | TGTTCTTCTTGTCTTGGGTA |
| NC-siRNA-sense | UUCUCCGAACGUGUCACGUTT |
| NC-siRNA-antisense | ACGUGACACGUUCGGAGAATT |
| HOXD10-SiR1-F | CCGAACAGAUCUUGUCGAATT |
| HOXD10-SiR1-R | UUCGACAAGAUCUGUUCGGTT |
| HOXD10-SiR2-F | GAUAAGCGCAACAAACUCATT |
| HOXD10-SiR2-R | UGAGUUUGUUGCGCUUAUCTT |
